# Etiology of community-acquired pneumonia in adults: a systematic review

**DOI:** 10.1186/s41479-020-00074-3

**Published:** 2020-10-05

**Authors:** Saeed Shoar, Daniel M. Musher

**Affiliations:** 1 Medical Care Line (Infectious Disease Section), Michael E. DeBakey Veterans Affairs Medical Center, 2002 Holcombe Boulevard, Room 4B-370, Houston, TX 77030 USA; 2 Department of Medicine, Baylor College of Medicine, Houston, TX 77030 USA

**Keywords:** Community acquired pneumonia, Etiology, Pneumococcus, Antibiotic stewardship, Guidelines for management of CAP

## Abstract

**Background:**

The etiology of community-acquired pneumonia (CAP) has evolved since the beginning of the antibiotic era. Recent guidelines encourage immediate empiric antibiotic treatment once a diagnosis of CAP is made. Concerns about treatment recommendations, on the one hand, and antibiotic stewardship, on the other, motivated this review of the medical literature on the etiology of CAP.

**Methods:**

We conducted a systematic review of English-language literature on the etiology of CAP using methods defined by the Preferred Reporting Items for Systematic Reviews and Meta-Analyses (PRISMA) guidelines. We searched PubMed using a combination of the keywords ‘pneumonia’, ‘CAP’, ‘etiology’, ‘microbiology’, ‘bacteriology’, and ‘pathogen’. We examined articles on antibiotics that were develop to treat pneumonia. We reviewed all ‘related articles’ as well as studies referenced by those that came up in the search. After we excluded articles that did not give sufficient microbiological data or failed to meet other predetermined criteria, 146 studies remained. Data were stratified into diagnostic categories according to the microbiologic studies that were done; results are presented as the percentage in each category of all cases in which an etiology was established.

**Results:**

*Streptococcus pneumoniae* remains the most common cause of CAP although declining in incidence; this decline has been greater in the US than elsewhere. *Haemophilus influenzae* is the second most common cause of CAP, followed by *Staphylococcus aureus* and Gram negative bacilli. The incidence of all bacteria as causes of CAP has declined because, with routine use of PCR for viruses, the denominator, cases with an established etiology, has increased. Viruses were reported on average in about 10% of cases, but recent PCR-based studies identified a respiratory virus in about 30% of cases of CAP, with substantial rates of viral/bacterial coinfection.

**Conclusion:**

The results of this study justify current guidelines for initial empiric treatment of CAP. With pneumococcus and *Haemophilus* continuing to predominate, efforts at antibiotic stewardship might be enhanced by greater attention to the routine use of sputum Gram stain and culture. Because viral/bacterial coinfection is relatively common, the identification of a virus by PCR does not, by itself, allow for discontinuation of the antibiotic therapy.

## Introduction

Community-acquired pneumonia (CAP) is the term used to describe an acute infection of the lungs that develops outside the hospital setting in a patient who has not been recently hospitalized. Reports in the medical literature have generally defined pneumonia as a new (although it should properly be stated as ‘newly recognized’) pulmonary infiltrate on chest X-ray or computerized tomography (CT) together with ≥ 2 of the following findings: new or worsening cough, sputum production or shortness of breath; pleuritic chest pain; fever or hypothermia; oxygen desaturation; confusion; leukocytosis or leukopenia [1–3]. Among elderly patients, CAP may present with less apparent symptoms [4, 5]. Until 2019, a commonly used definition of CAP excluded persons who had frequent contact with the healthcare system, such as those who were on hemodialysis or were admitted from nursing homes [1]; in such patients, a diagnosis of healthcare-associated pneumonia (HCAP) was made. These patients are now included in the definition of CAP [3].

CAP remains a major cause of morbidity and mortality in the United States, occurring in 649 [6] to 847 [7] adults per 100,000 population and causing around 1.6 million hospitalizations per year. McLaughlin et al. [8] stratified populations into adults < 65 and those ≥ 65 years of age; not surprisingly, the incidence of hospitalization for pneumonia was 10 times greater in the older than in the younger group (about 2000 vs. 200 per 100,000 per year). In 2017, 46,200 deaths were attributed to “influenza and pneumonia” (with no distinction between CAP and hospital-acquired pneumonia) [9]. The adjusted mean cost of hospitalization for a case of CAP, including complications and readmissions, was recently calculated at $55,000 [10], resulting in a total annual cost of $88 billion in the US. Using the above incidence data, the corrected total cost would be $110 billion.

CAP is a heterogeneous entity with a variable clinical presentation and a wide range of responsible pathogens that differ depending upon age, geography and recent exposures. In its deliberations, the committee that wrote the *2019 Official Clinical Practice Guideline of the American Thoracic Society and Infectious Diseases Society of America* [3] paid remarkably little attention to the etiology of CAP (Daniel Musher, personal communication). In light of these newly published guidelines for initial, empiric treatment of CAP on the one hand and increasing societal concern for antibiotic stewardship on the other, a review of etiology seems to be highly appropriate. To our knowledge this subject has not been reviewed in this fashion in recent years.

Such a review is far more complicated than it might appear to be. There is no question that the etiology of CAP has undergone a substantial evolution since the pre-antibiotic era [11]. *Streptococcus pneumoniae* (pneumococcus) is less frequently implicated, new agents have been identified, modern diagnostic techniques have been developed and the validity of some reported techniques has been questioned. Furthermore, despite diligent efforts to identify the cause, in most studies, an etiology has not been determined in one-half or more of the patients who are hospitalized for CAP [12–15]. Comprehensive molecular testing [16] or quantitative bacteriologic studies [17] may greatly increase the yield, but these techniques are not generally available, and the studies that utilized them were confined to patients who produced valid sputum samples, before prolonged antibiotic therapy or required intubation,probably no more than 50% of patients. We have systematically reviewed the medical literature on the etiology of CAP, stratifying articles in accord with the evolution of laboratory techniques for establishing microbiologic diagnoses.

## Methods

### Study design

A systematic review was conducted according to the Preferred Reporting Items for Systematic Reviews and Meta-Analyses (PRISMA) [18]. Two investigators independently performed the literature review and screened the articles for relevance and eligibility. Any conflict was resolved by consensus.

### Search strategy

We queried MEDLINE/PubMed from January 1945 until March 2020 to identify studies that reported the etiology of CAP using a combination of <pneumonia>, <CAP>, or < community acquired pneumonia> and < etiology>, <microbiology>, <bacteriology>, <cause>, <etiologic agent>, <causative agent>, or < pathogen> as search terms. We reviewed ‘related articles’ as well as articles referenced by those that came up in the search. We also searched by <pneumonia> and each of the many antibiotics that were developed for use in treating this infection in the past 6 decades. These included but were not limited to ciprofloxacin, moxifloxacin, ofloxacin, levofloxacin, delafloxacin, omadacycline, doxycycline, tigecycline, ceftriaxone, cefuroxime, clarithromycin, azithromycin, telithromycin**,** ampicillin/sulbactam, piperacillin/tazobactam, and ticarcillin/clavulanate,

We screened the titles and abstracts of articles for relevance and accessed the full-text of relevant articles for eligibility. The bibliographies of eligible articles were further examined for potentially relevant studies.

### Study selection

We confined this systematic review to original articles in the English language on confirmed cases of CAP in adults from developed countries (see https://isge2018.isgesociety.com/registration/list-of-developing-countries/. We only used articles that provided sufficient information to determine a microbial etiology through diagnostic laboratory techniques that were available at the time of the study. An eligible study was included in data synthesis only if the total number of cases and the numbers with confirmed causative agents were extractable and if an etiologic diagnosis was established in at least 25% of cases. We did not confine our study to hospitalized patients, although the great majority of subjects, in fact, were inpatients; thorough microbiologic studies are not usually done in outpatients.

We excluded studies that focused on a single pathogen or complication of pneumonia (such as empyema), studies that were performed during a specific outbreak such as the recent COVID-19 pandemic, or those that focused on a specific population, although we did not exclude studies of “elderly subjects” because the majority of patients who develop CAP are older adults. Case reports, review articles, editorials and commentaries were also excluded.

### Data abstraction

Date on the following variables were extracted from each article and listed in the study-designated spreadsheet (See Supplementary Data, Table 1): first author’s name, publication year, country of study population, study type, number of confirmed cases of CAP, study setting (inpatient versus outpatient), frequency of each diagnostic test performed, prior antibiotic exposure, number of patients with an identified microbiologic etiology, and the numbers of cases attributed to each organism.

### Outcome measures

The primary endpoint of this systematic review was the proportion of patients with each microbial cause, defined as the number of patients with that cause divided by the total cases of CAP for which a microbial etiology was determined. An important secondary endpoint was the proportion of all cases in which a microbial cause was determined.

### Data synthesis

For the purposes of reporting results, we stratified articles into categories based on the microbiologic techniques that were utilized. In a general way, these categories reflect the evolution of laboratory techniques in the past 75 years: (1) Standard cultures for bacteria, including cultures of sputum, transtracheal aspirates, bronchoscopic washings/brushings, as well as cultures of blood and other normally sterile secretions/fluids; (2) Bacterial cultures (as above in #1) plus cultures and/or serology for one or more so-called “atypical” organisms (*Mycoplasma, Chlamydia, Legionella* or rickettsiae); (3) Bacterial cultures plus cultures and/or serology for atypical organisms and viruses; and (4) Modern studies, which utilized most or all of the above techniques plus: (a) PCR for atypicals; (b) PCR for viruses; or (c) PCR for both atypicals and viruses. Some studies supplemented their microbiological assessment by urinary antigen testing for pneumococcus and/or *Legionella*.

Understanding potential problems related to various diagnostic techniques (see Discussion), we chose to accept and report all results as they were presented. Data for each of the categories listed above were pooled to determine the overall frequency of each etiologic agent within that category. Results for each individual study that was included in the final analysis are provided in Supplementary Data, Table 1.

## Results

**Fig. 1:**
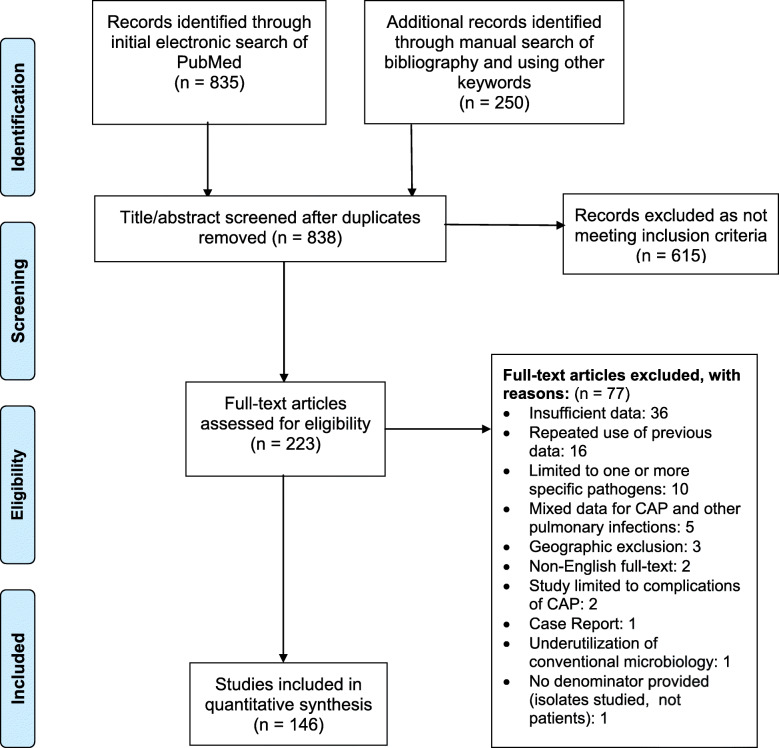
Flowchart of systematic literature review and study selection

Our literature search yielded a total of 1085 articles of which 838 remained after duplicates were removed. Title/abstract screening further excluded 615 articles that did not appear to be relevant. The full text of 223 relevant articles was reviewed in detail. Of these, 77 articles did not meet our predetermined eligibility criteria and were excluded (see flowsheet, Fig. 1), leaving 146 articles for inclusion in this systematic review.

The 146 papers that we included reported a total 82,674 patients with CAP, of whom 63,938 (77.3%) were inpatients, 16,532 (20.0%) were inpatients or outpatients, and 2204 (2.7%) were outpatients. The numbers of studies and patients and the stratification into microbiologic categories based on the diagnostic techniques are summarized in Table 1.

**Table 1:** Characteristics of studies reporting the etiology of community acquired pneumonia (CAP)

Nature of the microbiologic studies	Bacteria only	Bacteria & ‘atypicals’ ^a^	Bacteria, ‘atypicals’ & viruses	Modern Studies: Bacteria and:		
				PCR for ‘atypicals’	PCR for viruses	PCR for ‘atypicals’ and viruses
**Number of studies**	25	37	46	16	10	12
**Publication years**	1945–2010	1984–2020	1967–2017	1999–2020	2008–2019	2005–2019
**Study setting**						
Inpatient only	8653	14,281	23,555	6790	6260	4399
Inpatient/outpatient	118	3786	3512	1069	5295	2752
Outpatient only	610	1368	226	0	0	0
**Number of CAP patients**	9381	19,435	27,293	7859	11,555	7151
**Specifies antibiotic exposure prior to microbiologic testing**	687 (7.3%)	3104 (16%)	4203 (15.4%)	1074 (13.7%)	2085 (18%)	966 (13.5%)
**Number of patients with no etiology determined**	6293 (67.1%)	11,663 (60.0%)	13,704 (50.2%)	4484 (57.1%)	5823 (50.4%)	4380 (61.3%)

^a^ “Atypicals” is a term used loosely in publications to refer to *Mycoplasma, Chlamydophila, Legionella* and/or *Coxiella*

**Fig. 2:**
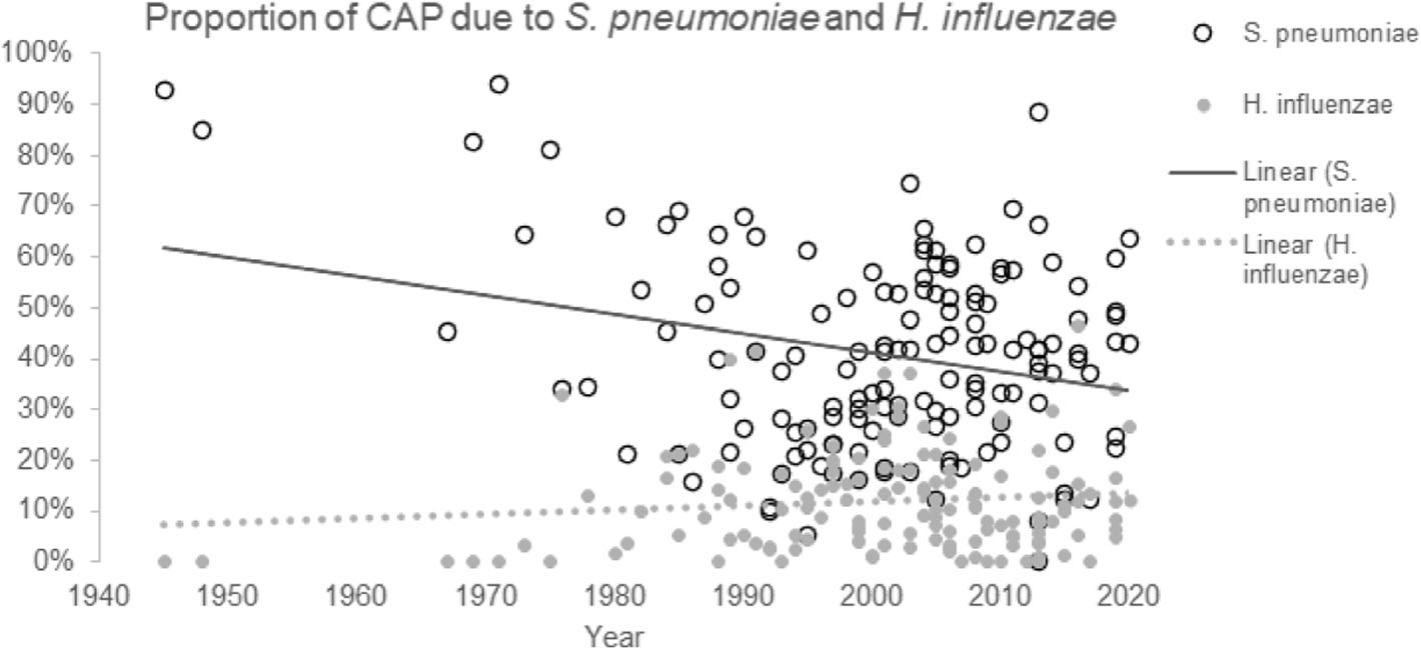
Trends of identification of *S. pneumoniae* and *H. influenzae* as the etiology of CAP

*Streptococcus pneumoniae* was the most common cause of CAP during the entire period and without regard to which microbiological technique were used, being identified, on average, in 33–50% of all cases in which an etiology was established (Table 2). The proportion of cases due to this organism appeared to decline with time (Fig. 2). *Haemophilus influenzae* was the second most common cause (7–16% of cases). *Staphylococcus aureus* and *Enterobacteriaceae* including *Klebsiella* were implicated with approximately equal frequency (4–10%). *Pseudomonas* (0.8–4.5%) and *Moraxella* (1.2–3.5%) were less common causes, and all other bacteria were isolated far less frequently. Among the so-called “atypical” bacteria, *Mycoplasma* caused about 4–11% of CAP, *Legionella* 3–8%, *Chlamydophila* 2–7% and *Coxiella* < 2%.

**Table 2:** Frequency of causative pathogens in CAP, stratified based on microbiologic techniques^a^

Studies were deigned to recognize:	Bacteria only	Bacteria & ‘atypicals’^b^	Bacteria, ‘atypicals’ & viruses	Modern Studies: Bacteria and:		
				PCR for ‘atypicals’	PCR for viruses	PCR ‘atypicals’ & viruses
*Streptococcus pneumoniae*	49.6	44.9	40.7	47.6	37.1	33.0
*Haemophilus influenzae*	13.8	14.0	9.7	15.6	7.2	8.6
*Haemophilus* (other)	3.3	3.8	0.2	3.1	0.1	0.1
*Moraxella catarrhalis*	1.7	2.0	1.2	3.5	2.2	2.4
*Staphylococcus aureus*	9.1	4.8	3.7	5.9	4.7	3.9
*Streptococcus pyogenes*	0.3	0.3	0.3	0.2	0	0.4
*Streptococcus* (other)	2.1	1.2	1.2	0.3	0.1	0.7
*Neisseria meningitidis*	0.3	0.04	0.1	0	0.02	0.0
*Klebsiella*	4.5	2.1	1.3	2.3	1.6	0.7
*Enterobacter*	0.4	0.4	0.2	0.2	0	0.0
*Enterobacteriaceae* (other)	4.8	4.1	2.6	2.0	2.8	2.7
*Pseudomonas*	2.4	2.2	2.9	1.9	4.5	0.8
Gram negative rods (other or unspecified)	3.0	2.3	3.3	1.8	0.5	1.8
Anaerobic bacteria	0.5	0.05	0.4	0.1	0	0.1
Other bacteria^c^	1.5	3.7	1.1	6.2	5.7	0.3
*Mycobacteria*	-- ^d^	0.5	1.04	0.3	0.03	1.8
*Pneumocystis*	–	0.3	1.1	1.2	0.1	0.2
Other fungi	–	0.01	0.3	0.1	0.02	0
*Nocardia*	–	0.03	0.05	0.03	0.03	0.04
Unspecified atypicals	–	0.5	0.4	0.0	0	0
*Mycoplasma pneumoniae*	–	8.8	10.0	10.5	3.7	8.9
*Chlamydophila pneumoniae*	–	6.9	5.7	4.9	1.4	2.9
*Chlamydophila* (other)	–	0.3	1.04	0.4	0.2	0.2
*Legionella*	–	8.3	6.5	6.6	3.3	6.2
*Coxiella*	–	0.3	1.8	0.6	0.5	0.3
Influenza A virus	–	–	2.4	0.1	4.7	3.4
Influenza B	–	–	0.8	0.0	0.3	1.1
Influenza A or B	–	–	1.3	0.3	1.2	9.2
Parainfluenza virus	–	–	1.0	–	0.8	4.6
Respiratory syncytial virus	–	–	0.8	–	1.5	4.7
Rhinovirus	–	–	0.04	–	4.1	11.5
Human metapneumovirus	–	–	0	–	0.4	4.1
Coronavirus	–	–	0	–	0.3	3.2
Bocavirus	–	–	0	–	0	0.04
Herpes simplex virus	–	0.01	0.3	–	0	0.1
Cytomegalovirus	–	–	0.1	–	0.02	0
Adenovirus	–	–	0.5	–	0.3	2.2
Varicella	–	0.03	0.2	–	0	0
Virus (unspecified or other)	1.04	0.03	1.8	0.1	9.6	0.6
Bacterial/viral coinfection	1.9	1.9	6.1	2.6	9.7	5.9

^a^ Results for each organism are reported as percentage of all patients for whom an etiology was determined

^b^ “Atypicals” is a term used loosely in publications to refer to *Mycoplasma, Chlamydophila, Legionella* and/or *Coxiella*

^c^ “Other bacteria” were listed as “other” in original publications or < 0.2% in every column

^d^ --, Not reported

Using data only from the last two categories in which PCR was routinely studied for viruses, influenza viruses were identified in 6.2–13.7% of cases and rhinoviruses in 4.1–11.5%. Respiratory syncytial virus and human metapneumovirus were less common (0.4–4.7%), followed more distantly by other viruses. In studies in which viral PCR was most consistently done [12, 13, 17], a respiratory virus was identified in 30 to 40% of patients, and bacterial/viral coinfection was found in 25–35% of these cases. These results were supported by a separate study [19] which looked at all viral illness to determine what proportion had bacterial coinfection; the reported rate of coinfection was 40%.

On average, no matter which category of microbiologic testing was done, no etiologic agent for CAP could be identified in more than one-half of cases (50.2–67.1%). Even with the addition of more sophisticated techniques in the past 2 decades, including routine utilization of PCR to identify atypical bacteria and viruses, there was no tendency toward an increased identification of a pathogen. Two recent studies, however, that did quantitative analyses using all current techniques but only included patients who provided high-quality sputum samples, were able to establish a final diagnosis in 87-95% of cases [16, 17].  

## Discussion

**Fig. 3:**
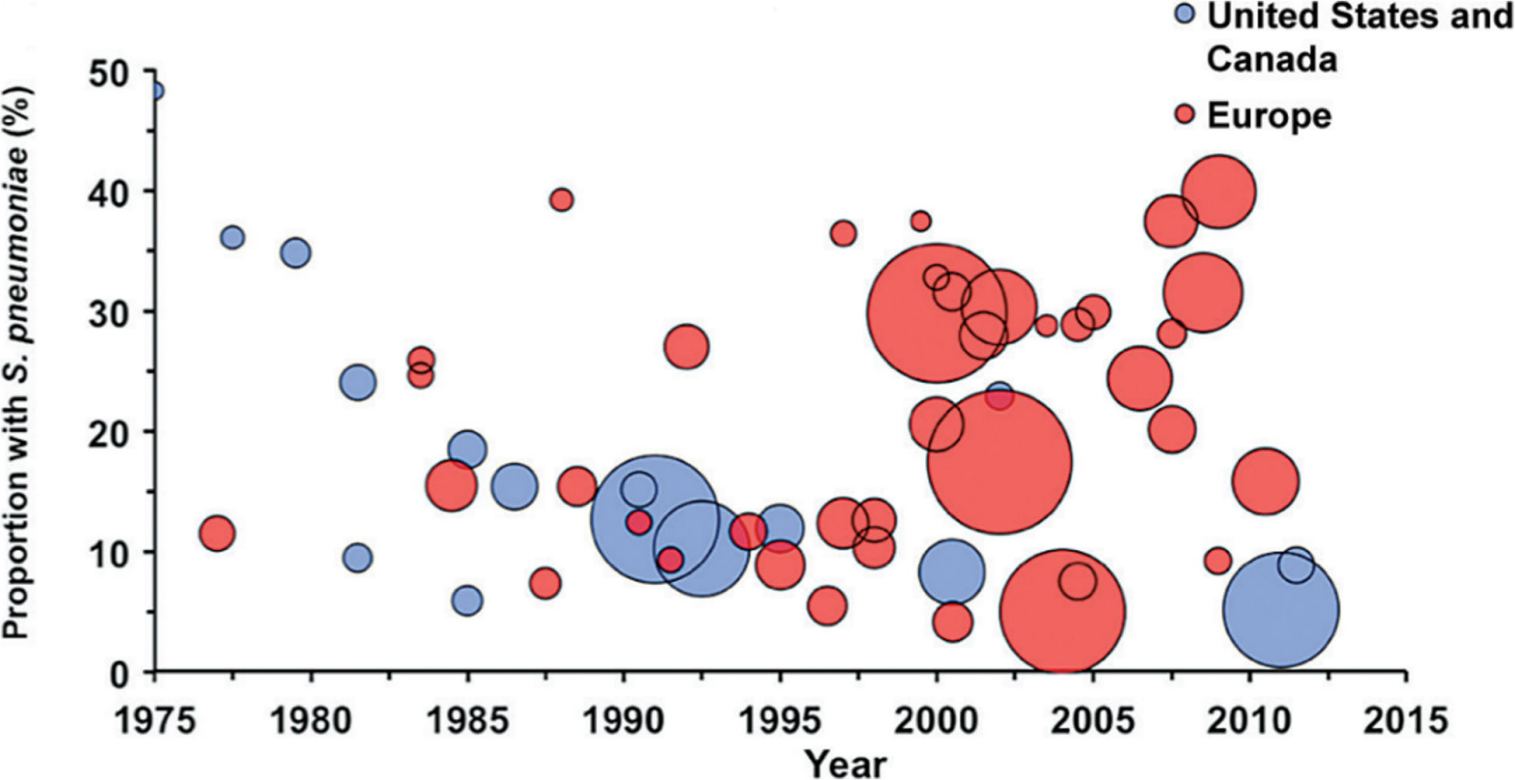
The proportion of *S. pneumoniae* as the causes of CAP in US and Canada vs. all other geographic regions (reprinted with permission from Musher et al. [11])

In the preantibiotic era, pneumococcus caused 90–95% of all pneumonias [11, 20, 21]; in fact, even into the 1960’s, ‘pneumococcal pneumonia’ and ‘pneumonia’ were almost regarded as synonymous [22]. Since the beginning of the antibiotic era, the proportion of CAP attributable to *S. pneumoniae* has steadily declined [11]. This decline has been much more pronounced in the US than in Europe [11–14, 23–25] (Fig. 3), a difference that might be explained by the widespread acceptance of pneumococcal vaccine in US adults (nearly two-thirds of eligible adults in the US have been vaccinated [26] compared to 20–30% in most European countries [27]), decreased rates of cigarette smoking in the US [28], the rapid implementation of recommendations to administer conjugate pneumococcal vaccine early in childhood and the early use of empiric antibiotics in outpatients. Part of this decline also reflects the larger denominator (cases in which another etiology was established); the routine use of PCR has increased the size of the denominator by identifying patients with viral pneumonia.

Methodologic considerations are far more important in determining the etiology of CAP than is often realized. The principal reasons for failing to diagnose bacterial pneumonia are: (1) the failure to obtain an adequate sputum sample for culture; and (2) obtaining a sputum after antibiotics have already been given, for example, ≥ 24 h [29]. Blood cultures are positive in only about 25% of cases of pneumococcal pneumonia [30, 31], and urine antigen might identify another 11–23% [31, 32], but blood cultures are positive less frequently in *Haemophilus* pneumonia [33] and almost never positive in *Moraxella* pneumonia [34]. In the absence of a positive culture of blood or pleural fluid, the only generally available way to identify bacteria as a cause of CAP is by sputum culture. These observations suggest that many or most of the undiagnosed cases of CAP are bacterial in origin.

Two studies that only included data from patients who provided a high-quality sputum sample support this concept; the investigators used entirely different techniques but came to similar conclusions that differ strikingly from most of the studies included in this systematic review. Using highly standardized quantitative PCR technology [35], Gadsby et al. [16] found an etiologic agent in 87% of cases, with pneumococcus, *Haemophilus* and *Moraxella* in 26, 40 and 14%, respectively. Musher et al. [17] used quantitative bacteriology on sputum of patients who had not received antibiotics for more than 16 h (66% for ≤ 2 h) and identified a causative organism in 95.6% of cases, with pneumococcus, *Haemophilus* and *Moraxella* in 22, 22.5 and 15%, respectively. The essential difference between their studies and others reported previously is their dependence upon good-quality sputum. Importantly, Musher et al. [17] excluded patients who had received > 16 h of antibiotics, and two-thirds of their patients had received ≤ 2 h of antibiotic therapy. (Since Gadsby et al. [16] were using PCR technology, the viability of bacteria was not an issue, and prior antibiotic administration did not matter.) Musher et al. also reported that bacteria which are generally regarded as “normal respiratory flora” (*Streptococcus mitis, Corynebacteria* and other oral streptococci) caused about 20% of all cases of CAP, either alone or together with a respiratory virus, further implicating bacteria as etiologic agents of CAP. Each of these two studies identified a respiratory virus in about one-third of cases with viral/bacterial coinfection being common. Occasional earlier reports that confined themselves to good-quality sputum also had much higher diagnostic yields [29, 36, 37].

We chose to present our results as a proportion of all cases in which an etiologic agent was identified, understanding that whichever denominator is chosen, potential methodologic problems result. On the one hand, if no etiologic agent was identified, it could have been because some other, unidentified organism was responsible or, more likely, that sputum specimens had simply not been submitted or were only submitted after patients had received prolonged courses of antibiotics. To use the total CAP population as a denominator would greatly dilute all positive results. Using this approach, the important prospective EPIC study by the CDC identified *S. pneumoniae* in only 5% of cases of CAP, which simply does not adequately describe the situation [13]. Even our own prospective study [17] found pneumococcus in only 9% of all cases of CAP, because more than one-third of patients could not provide a valid sputum sample in a timely fashion. To report proportions when the denominator is simply unknown seemed to us to be entirely misleading. Thus, we chose to stratify our results based on diagnostic techniques that were available and the percentage of cases in which they were used. For our final denominator, we then used all cases in which an etiology was positively identified.

Reporting bias also leads to difficulties in interpreting literature on CAP. Case series of patients with CAP generally include all persons with this infection (although they tend to exclude immunocompromised hosts); however, the microbiologic data are limited because these studies are retrospective and, in the absence of a protocol to carry out microbiology studies, they are often not done. In contrast, reports of new antimicrobial agents present extensive microbiologic data because such results are required for approval by government agencies, but these studies nearly always excluded immunocompromised hosts [38], however defined, and are often biased by their exclusion of patients with severe illness. Even these studies may fail to determine an etiology in nearly one-half of cases [14].

We have regarded the diagnosis of “atypical” bacteria as problematic [2]. The frequency with which atypical agents are identified is especially dependent upon the age of the patient population and the methods used for diagnosis [13, 39]. In early studies of pneumonia due to *Mycoplasma pneumoniae,* when cultures and rigorous serologic studies were done, *Mycoplasma* was shown to be a very uncommon cause of pneumonia in older patients [40]. In fact, in a recent PCR-based study, Diaz et al. showed that, of all patients hospitalized for CAP due to *M. pneumoniae*, only 6.9% were > 50 years old and 3.9% were > 65 [41]. Pneumonia due to *Chlamydophila* was originally described in young adults [42, 43], although this organism may cause pneumonia leading to hospitalization of older patients [42]. Serologic diagnosis of pneumonia due to these organisms is problematic [44]. Although, for the purposes of this review, we did not dispute serologic diagnoses of “atypical pneumonias,” some were probably unacceptable by reasonable scientific standards. For example, one recent study [15] that attributed 20.4% of CAP to “atypicals,” defined a positive serologic result as: “a positive baseline or post-treatment evaluation IgM serologic test result, a negative baseline and indeterminate post-treatment evaluation IgG serologic test result, or a negative baseline and positive post-treatment evaluation IgG serologic test result [with no titers stated].” ELISA for IgM is notoriously unreliable, especially at the lower end of the readings, and an intermediate result is not the same as a positive result. However, the study by File et al. [14] that used rigorous diagnostic techniques identified atypical agents in an even higher percentage of patients; the average age of patients in that study was 56.5 years. In contrast, two European studies [45, 46] that accepted only a four-fold rise in IgG found atypical agents in 3.9 to 7.1% of CAP. Data from studies that utilized PCR on a nasopharyngeal swab in every case attributed a similarly small percentage (3.3%) to atypicals [13]. We are unable to resolve these discrepancies.

### Limitations

The principal limitation of this systematic review is the extraordinary heterogeneity of the microbiologic data. For this reason, we attempted to stratify those studies that we included based on the microbiologic analyses that were done, an approach that might have reduced, but certainly did not eliminate, the heterogeneity problem. Most publications did not state the proportion of cases in which samples were submitted for microbiologic evaluation, and gave little insight into the nature or duration of antibiotic administration before sputum samples were obtained. Furthermore, when several methods were used to reach an etiologic diagnosis (for example, serology and PCR for *Chlamydophila*), results were sometimes reported as the proportion positive for each test, and it was not possible to determine what number of patients had documented positive test results for each method. Similarly, some studies stated the frequency with which an etiologic agent was found by blood culture and the frequency with which it was found by sputum culture but did not specify potential overlap between these data, in which situation we had to use an estimation to calculate the data pertinent to each etiologic pathogen. Papers sometimes reported data based on numbers of isolates, not numbers of patients; in cases of coinfection, such reporting inflated the proportion of patients who had bacterial pneumonia. Finally, different denominators were often used for the percentage of each causative pathogen based on the diagnostic method utilized to document that agent.

## Conclusions

The results of this systematic review show that pneumococcus and *Haemophilus* continue to predominate as the bacterial causes of CAP, followed by *Staphylococcus aureus* and *Enterobacteriaceae*. For all the emphasis on *Pseudomonas,* this organism remained a relatively uncommon cause. On average, *Moraxella* was implicated in 2–3% of cases, although some series showed this organism to be the third most common cause of CAP, following pneumococcus and *Haemophilus*. Other bacteria were isolated far less frequently. In nearly all reports, an etiology could not be identified in more than one-half of cases. Studies that were confined to patients who provided good-quality sputum reported substantially higher yields for bacterial causes. One study called attention to the role of so-called normal respiratory flora as a prominent cause of CAP. We were unable to resolve wide discrepancies among individual studies that reported *Mycoplasma* and *Chlamydophila* in 4–20% of cases (average about 10–15%). On average, viruses were found in about 10% of case series, but recent reports that included viral PCR on most or all patients found a respiratory virus in about 30% of cases, with bacterial/viral coinfection being relatively common.

These results appear to support the 2019 guidelines for the initial empiric management of CAP [3] that recommend ceftriaxone and azithromycin as therapy for hospitalized patients because of the expected efficacy against pneumococcus, *Haemophilus*, *Staphylococcus aureus* and *Enterobacteriaceae*, as well as “atypical” organisms. The finding of a virus by PCR does not eliminate the need to treat empirically for bacteria, because bacterial/viral coinfection is relatively common. More careful attention to sputum Gram stain and culture results should improve outcomes in patients who do not respond to initial empiric therapy and might enable better antibiotic stewardship. Molecular techniques may, in the future, simplify the process of correct identification of causative organisms.

## Supplementary information

**Additional file 1.**

## Data Availability

This is a systematic literature review for which extracted data is published in supplementary Table 1.
